# A slow growing elastic papulonodular lesion

**DOI:** 10.1016/j.jdcr.2025.01.028

**Published:** 2025-02-24

**Authors:** María Jesús Barros Eyzaguirre, Nicolas Silvestre-Torner, Fernando Gruber Velasco, Belén Romero Jiménez, Catalina Axpe Gil, Adrián Imbernón Moya, Marcela Martínez Pérez, Gonzalo Garcia de Casasola Rodriguez

**Affiliations:** a Department of Dermatology, Hospital Universitario Severo Ochoa, Avenida de Orellana, Leganés, Madrid, Spain; b Department of Pathology, Hospital Universitario Severo Ochoa, Avenida de Orellana, Leganés, Madrid, Spain

**Keywords:** high-resolution skin ultrasound, histology, nodulocystic basal cell carcinoma

## Clinical case

**Fig 1:**
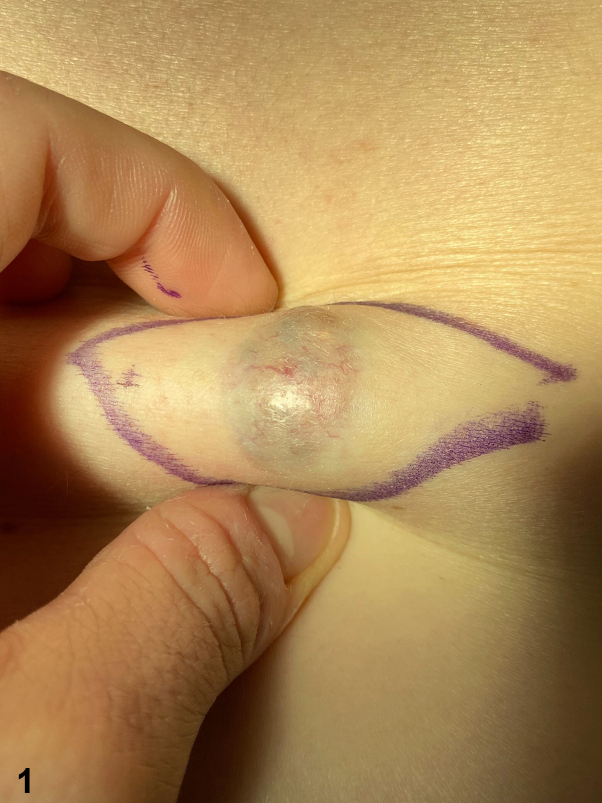

**Fig 2:**
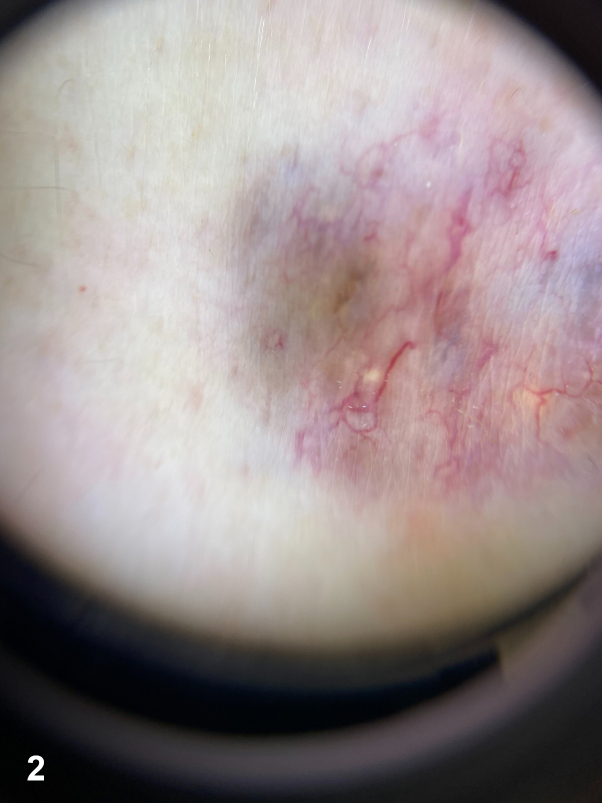

**Fig 3:**
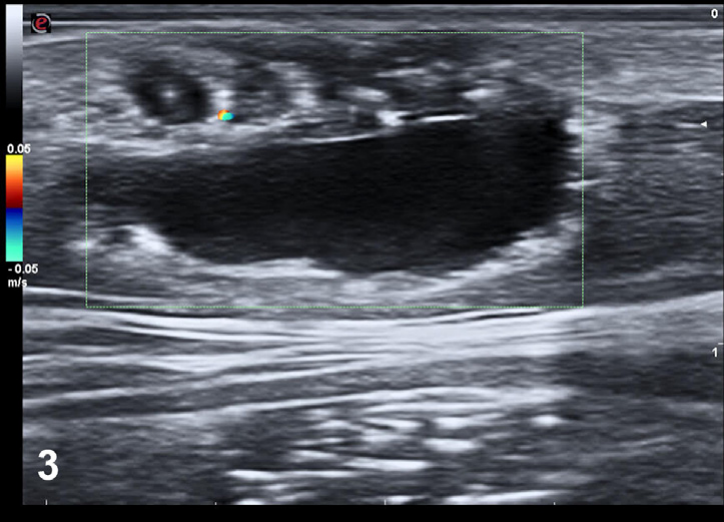

**Fig 4:**
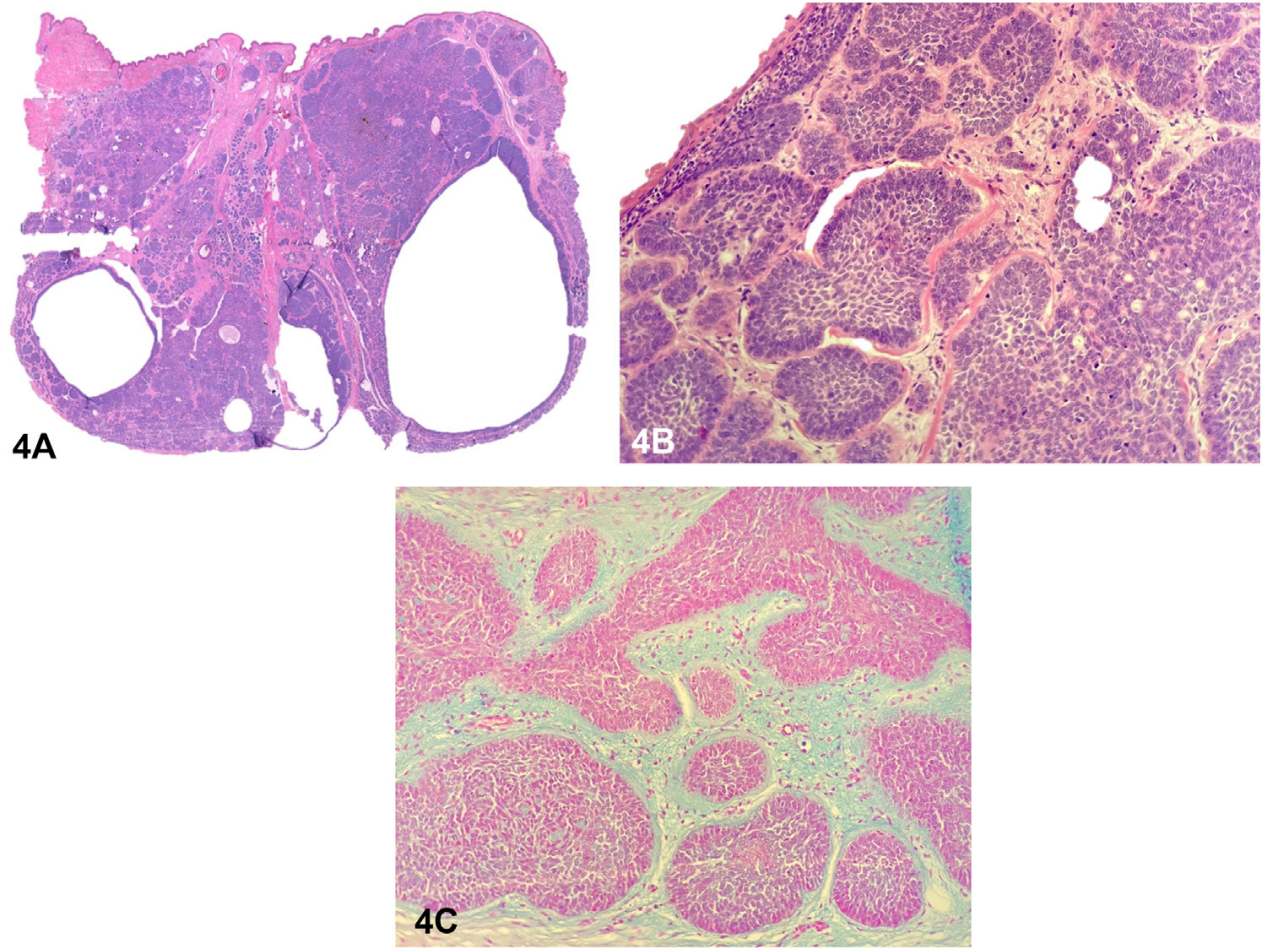

A 45-year-old woman presented with a solitary, asymptomatic, slightly elevated violaceous papulonodular lesion with cystic appearance that appeared on her right lumbar region 16 years ago. On examination, it had an elastic and soft consistency (Fig 1). Dermoscopic examination revealed arboriform telangiectasias over a bluish and reddish diffuse area (Fig 2). High-resolution skin ultrasound found a well-defined heterochoic nodule in dermis and hypodermis with negative Doppler signal (Fig 3). Histologic examination showed a lesion in dermis that reaches the papillary and reticular dermis but with no epidermal connection and is formed by basaloid cells forming a palisade in the peripheral cell row (Fig 4, *A*-*C*).

**Question 1: What is the most likely diagnosis?**

A. Pilomatrixoma
B. Trichoepithelioma
C. Cylindroma
D. Nodulocystic basal cell carcinoma
E. Spiradenoma

**Answers:**

A. Pilomatrixoma – Incorrect. Pilomatrixoma is a benign adnexal tumor derived from hair follicle structures. It usually presents in children as a single, firm nodule.^1^ Histologically, the tumor is lobulated and it is sometimes surrounded by a pseudocapsule. Ghost/shadow cells may predominate.
B. Trichoepithelioma – Incorrect. Trichoepithelioma is a benign tumor derived from hair follicle. It presents as a generally solitary small annular papular lesion with indurated consistency and depressed center.^1^ Histologically, this lesion is made up of cords and nests of basaloid cells that can connect with the epidermis and the follicular infundibulum. It can be intermingled into small infundibular cysts.^2^
C. Cylindroma – Incorrect. Cylindroma is a benign tumor derived from apocrine cells in dermis. It presents as a slowly growing solitary pink or red nodule found on the scalp or face.^1^ On histopathology, it is classically described ad islands or multiple lobules of basaloid cells that arranged together in a compact jigsaw pattern.^2^
D. Nodulocystic basal cell carcinoma – Correct. Nodulocystic basal cell carcinoma (BCC) is a rare subtype of nodular BCC in which tumor necrosis leads to cavitation and cystic formation. It presents as a slowly growing nodule with cyst consistency. On histology, it shows basaloid cells with peripheral palisading and variable–sized cysts.^2^
E. Spiradenoma – Incorrect. Spiroadenoma is a benign adnexal tumor that commonly presents as a small bluish-pink to gray subcutaneous nodule, usually in the upper half of the body, typically painful.^1^ Histologically, it is composed of intradermal lobes surrounded by a fibrous capsule without connections with the epidermis. Epithelial cells within the tumor lobe are arranged in interlocking cords.^2^

**Question 2: What type of material is seen inside the lesion?**

A. Keratin
B. Mucin
C. Hyaline material
D. Amyloid
E. Hemosiderin

**Answers:**

A. Keratin – Incorrect. Keratin is the protein that results from cutaneous epithelial cell differentiation. It can be found inside epidermal inclusion cysts or in well differentiated squamous cell carcinoma. Immunostaining is used to identify keratin produced by epithelial cells.^3^
B. Mucin – Correct. Mucin is a glycoprotein produced by various epithelia, including gastrointestinal, respiratory and reproductive tract. Mucin is also seen in the stroma of nodulocystic BCC. Alcian blue is one of the classic stains used to demonstrate the presence of mucin.^3^
C. Hyaline material – Incorrect. Hyaline is a pale, glassy, acellular, unstructured proteinaceous material that results from epithelial injury and amounts of soluble and insoluble proteins. It stains eosinophilic with hematoxylin and eosin stain or bright pink with Periodic Acid-Schiff staining.^3^
D. Amyloid – Incorrect. Amyloid appears from aggregates of proteins characterized by a fibrillar morphology. It has no structural function and is associated with a group of diseases known as amyloidosis. It stains with Congo red and shows a characteristic apple-green birefringence when visualized by light microscopy.^3^
E. Hemosiderin – Incorrect. Hemosiderin is a yellow-brown pigment with granular or crystalline appearance derived from hemoglobin. It can be detected with Perls’ Prussian blue staining.^3^

**Question 3: Which of the following ultrasound findings is seen in this case?**

A. Intralesional hyperechoic dots with posterior acoustic shadow
B. Intralesional hyperechoic dots without posterior acoustic shadow
C. Linear echogenic lines at right angles to the ultrasound beam
D. Posterior enhancement.
E. Side shadows

**Answers:**

A. Intralesional hyperechoic dots with posterior acoustic shadow – Incorrect. They are typically found in pilomatrixomas and correlate with intralesional calcifications. They are produced because of the collision of the ultrasound beams with a material that is highly reflective and attenuates more than adjacent tissue.^4^
B. Intralesional hyperechoic dots without posterior acoustic shadow – Correct. They are considered a characteristic finding of BCC. The significance of these dots is unknown, and they don't correspond to calcifications or necrotic areas in histological preparations.^4^
C. Linear echogenic lines at right angles to the ultrasound beam – Incorrect. It is a sign produced by internal reflections. It is often found when examining lipomas.^4^
D. Posterior enhancement – Incorrect. Acoustic enhancement also called posterior enhancement is an ultrasound artifact seen when the ultrasound echoes pass almost completely through a watery material without reverberation, reaching the tissue behind the material and forming a hyperechogenic halo. On skin examination, it can be found in simple cysts.^4^
E. Side shadows – Incorrect. It occurs when the beam hits curved surfaces that separate media with different acoustic impedances. They usually occur in epidermal inclusion cysts.^4^

## Conflicts of interest

None disclosed.
